# Management of Postoperative Hematoma Following a Lingual Frenectomy: A Case Report

**DOI:** 10.7759/cureus.78363

**Published:** 2025-02-01

**Authors:** Bhavana J Jadhav, Siddhartha Varma, Girish Suragimath, Sameer A Zope, Nilesh Mishra, Harshvardhan A Mohite

**Affiliations:** 1 Periodontology, School of Dental Sciences, Krishna Vishwa Vidyapeeth, Karad, IND; 2 Oral and Maxillofacial Surgery, School of Dental Sciences, Krishna Vishwa Vidyapeeth, Karad, IND; 3 Pedaitric and Preventive Dentistry, School of Dental Sciences, Krishna Vishwa Vidyapeeth, Karad, IND

**Keywords:** ankyloglossia, complications, hematoma, lingual frenectomy, lingual frenum

## Abstract

Ankyloglossia, commonly known as tongue-tie, is a congenital condition characterized by a short, thick lingual frenum that restricts tongue mobility, leading to functional and developmental issues. Surgical intervention, such as a lingual frenectomy, is commonly used to restore tongue function. However, postoperative complications like hematomas can pose significant risks. This case report discusses the management of a sublingual hematoma in a 15-year-old male following a conventional lingual frenectomy. Postoperatively, the patient developed swelling, erythema, and restricted tongue movement, later diagnosed as a hematoma in the sublingual space through ultrasonography. Immediately, the patient was transferred to the critical care unit and started on intravenous antibiotics and analgesics, magnesium sulfate dressing, and close monitoring to prevent airway obstruction The patient improved significantly within 48 hours and completely recovered by the seventh day. This report underscores the importance of early recognition of complications, and prompt management to alleviate risks and ensure favorable outcomes. Furthermore, the necessity for better documentation and the use of multidisciplinary approaches in the management of ankyloglossia is emphasized.

## Introduction

Ankyloglossia, or tongue-tie, is a congenital condition characterized by a short, thick lingual frenum that restricts tongue movement [1]. In 1960, Wallace defined tongue-tie as a condition where the tongue tip cannot protrude beyond the lower incisor teeth because of a short frenulum lingua [2]. Ankyloglossia affects about 5% of infants, leading to breastfeeding difficulties, choking, and speech delays, particularly with consonants like *t*, *d*, *n*, and *l*. Ankyloglossia children may also develop habits like mouth breathing. Ankyloglossia increases the risk of class III dental malocclusions and issues with facial growth. Surgical correction is often necessary and typically involves a frenotomy or frenectomy, during which an incision is made using a scalpel, electrocautery, or laser [3]. Although generally straightforward, this surgery can lead to complications such as excessive bleeding, retention cysts, sublingual hematomas, infections, recurrence of frenal attachment, and speech disorders. The most common issues are the recurrence of frenal attachment and restricted tongue movement [4].

Substantial literature exists on techniques for addressing ankyloglossia; however, few researchers have explored the complications associated with lingual frenectomy and its management. This case report aims to shed light on the postoperative complications of sublingual hematoma following a lingual frenectomy and its management.

## Case presentation

**Figure 1:**
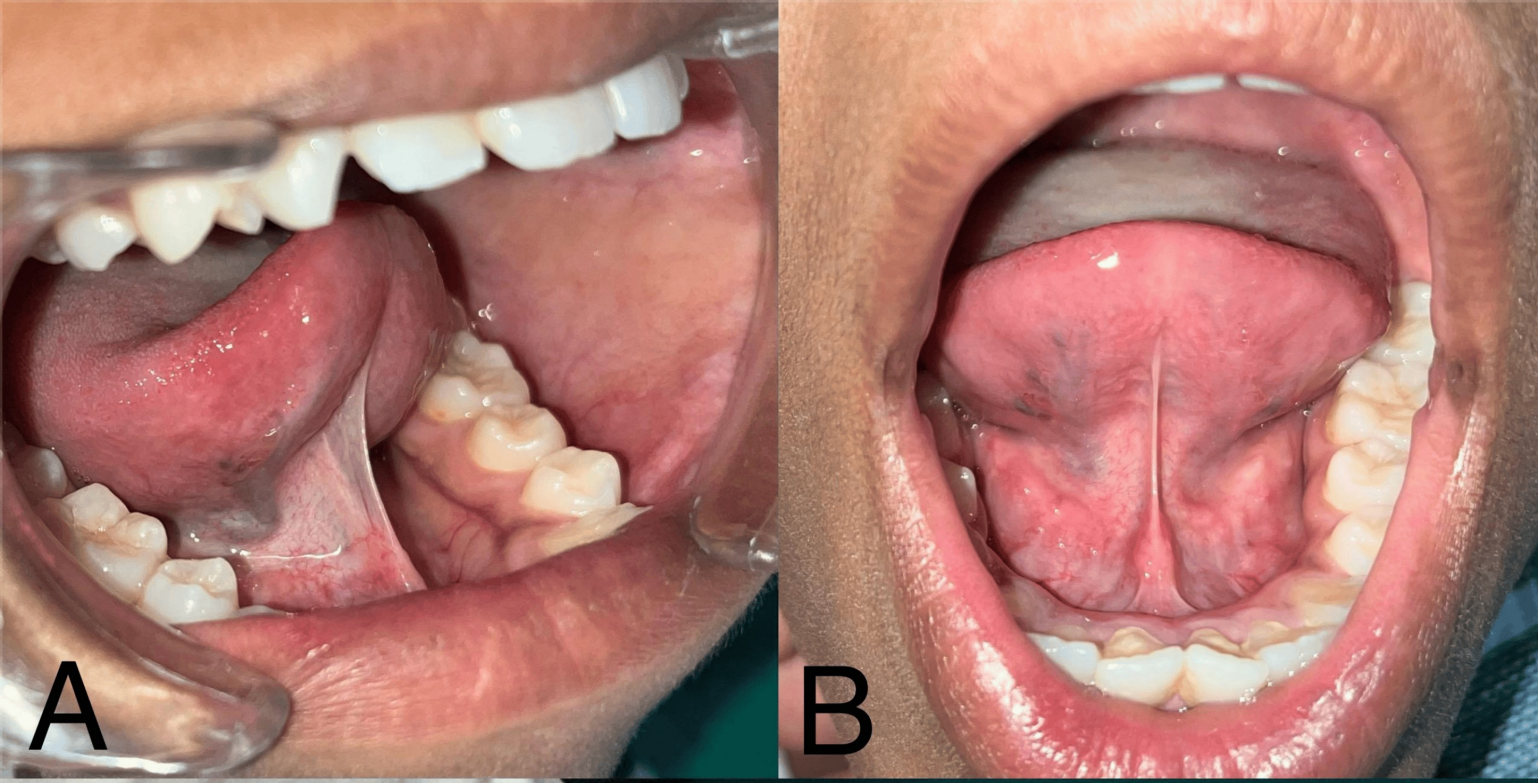
Preoperative intraoral view of ankyloglossia: (A) lateral view and (B) frontal view.

A 15-year-old male patient presented to the School of Dental Sciences, Krishna Vishwa Vidyapeeth, with a primary complaint of restricted tongue movement and difficulty in speech. The extraoral examination revealed no pathological abnormalities. The patient was systemically healthy; this was his first visit to the dentist. The patient was conscious and cooperative, with all vital signs within the normal range. Intraoral examination revealed limited tongue protrusion and lifting of the tongue tip due to the fusion of the lingual frenulum, characterized by a thick, short frenulum (Figures 1A, 1B). The patient was diagnosed with class II ankyloglossia according to Kotlow’s classification (Table 1) [5]. During the initial appointment, the patient was advised to undergo routine blood investigations, and all the values were within the normal range.

**Table 1:** Kotlow’s classification of ankyloglossia. Source: [5].

Class of ankyloglossia		Range
Class I	Mild ankyloglossia	12-16 mm
Class II	Moderate ankyloglossia	8-11 mm
Class III	Severe ankyloglossia	3-7 mm
Class IV	Complete ankyloglossia	<3 mm

**Figure 2:**
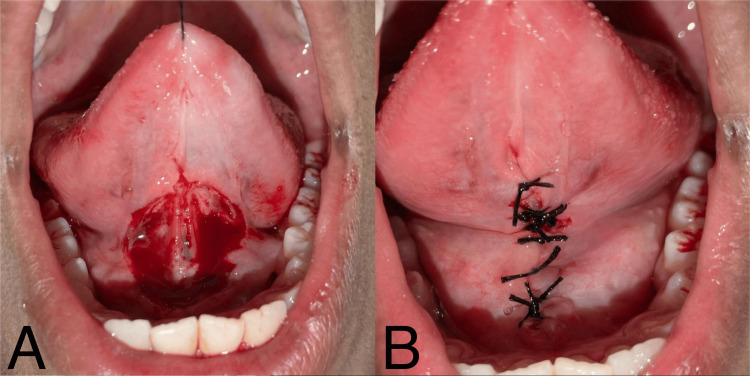
Surgical view: (A) lingual frenectomy performed with a scalpel and blade and (B) non-resorbable silk sutures (3-0) placed.

The parent and patient were informed about the treatment plan and consent was obtained. The treatment plan included a conventional surgical lingual frenectomy with a scalpel and blade. In the second appointment, a lingual frenectomy was performed under local anesthesia. The conventional/classical frenectomy technique was performed as explained by Archer [6] and Kruger [7]. Local anesthesia was achieved by administering 2% lignocaine (Easycainne® 2% Adrenaline, 1:200000, Naprod Life Sciences Pvt. Ltd., Mumbai, India). A silk suture was passed through the tip of the tongue and held by the operator to immobilize the tongue during the procedure. The frenum was engaged with a hemostat. Incisions were made over and under the hemostat until the frenum was freed, and the tissue was retracted using the hemostat. Fibrous attachments were separated, and the tongue was made completely free from the underlying attachment to the base of the tongue (Figure 2A). Non-resorbable silk sutures (3-0) were used to approximate the wound edges completely (Figure 2B). Oral amoxicillin 500 mg thrice a day and paracetamol 500 mg twice a day for five days were prescribed. The patient was instructed to eat a soft diet, avoid hot or spicy foods, apply ice packs to reduce swelling, gently clean the area, and perform prescribed tongue exercises to prevent reattachment. The patient was told to contact the operating doctor in case of any untoward incidents like excessive bleeding or severe pain.

**Figure 3:**
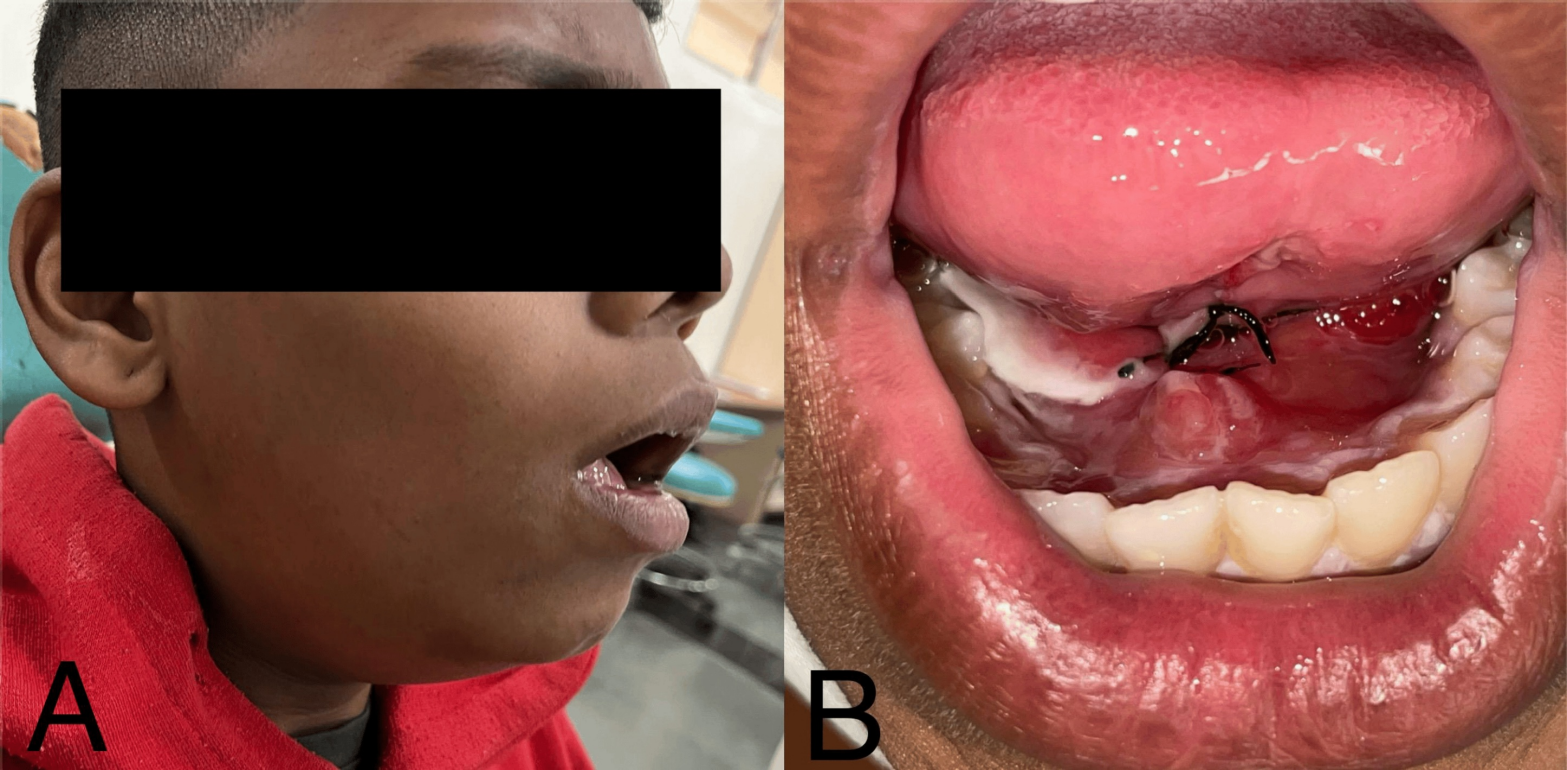
Postoperative day 2 view: (A) extraoral swelling on the lower right side of the face, extending from the angle of the mandible to the mid-chin region; (B) intraorally, edema, erythema, and induration were observed.

The patient reported to the clinic after two days with extraoral swelling on the lower right side of the face, difficulty in swallowing, reduced mouth opening, and restricted tongue movements. Swelling extended from the angle of the mandible to the mid-chin region on the right side of the face (Figure 3A). Intraorally, edema, erythema, and induration were observed (Figure 3B). He was promptly transferred to the critical care unit to monitor his vital signs. An ultrasonography (USG) of the submandibular region was performed, which showed edema in the subcutaneous region under the surgical site, with mild peripheral vascularity near the right submandibular gland, suggestive of hematoma of sublingual space. The patient was admitted to the intensive care unit to avoid the airway obstruction complications due to an increase in the size of hematoma.

**Figure 4:**
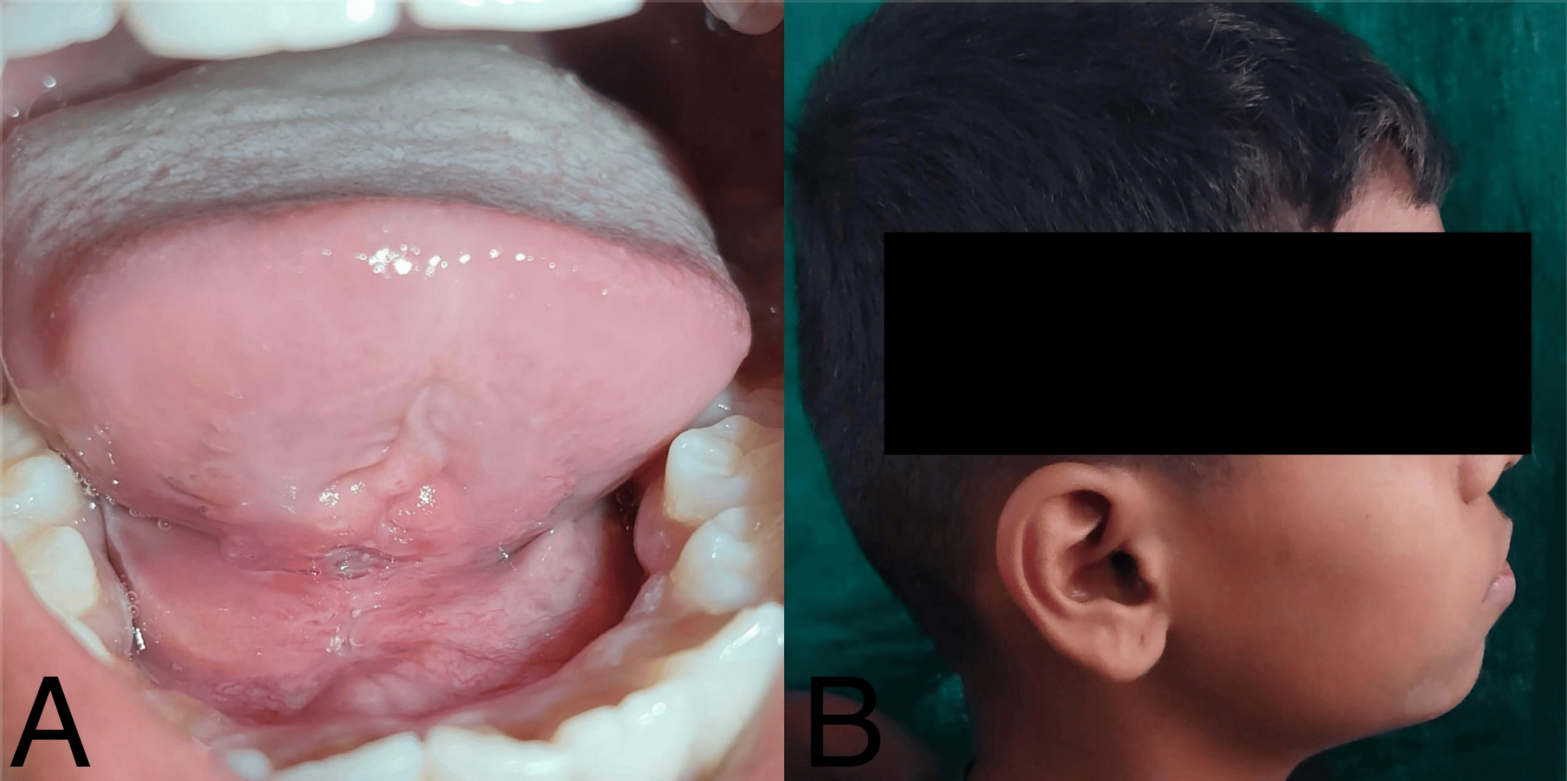
Follow-up after one week reveals complete resolution of hematoma symptoms.

The patient was administered an intravenous injection of Augmentin 1.2 g stat followed by 600 mg every 12 hours and an injection of Metronidazole 500 mg every eight hours to prevent the possibility of secondary infection. Magnesium sulfate dressing was applied extra orally to reduce the pain and swelling. Within the first 24 hours of initiating the medication, a significant reduction in the swelling was observed. After 48 hours, the intraoral edema, erythema, and induration had resolved by approximately 80%, enabling the patient to swallow and speak comfortably. On the third day, the patient was discharged with instructions to contact the doctor in case of any pain or discomfort. Intravenous antibiotics were stopped, and oral Augmentin 625 mg and Paracetamol 500 mg were prescribed twice daily for five days. The patient was recalled for a follow-up visit one week later, and the patient exhibited complete resolution of symptoms of hematoma (Figures 4A, 4B). Suture removal was carried out, and chlorhexidine mouthwash was advised for 15 days.

## Discussion

Before birth, a strong cord of tissue guides the development of the oral frenulum at the center of the mouth. Postnatally, the lingual frenulum influences tooth positioning and typically recedes and thins as the child develops. In some cases, the frenulum remains tight or fails to recede, causing tongue immobility - a condition called ankyloglossia, characterized by a short lingual frenulum that attaches the tongue to the floor of the mouth. The exact cause of ankyloglossia remains unknown [8]. Tongue-tie can cause tongue thrusting against the mandible, leading to mandibular prognathism and gingival recession on the lingual surfaces. Restricted tongue movement may result in improper chewing, swallowing, gastric distress, bloating, snoring, bedwetting, and difficulty with speech and activities requiring tongue gestures. It also increases the risk of dental caries due to impaired tongue cleaning and saliva distribution [9]. Ankyloglossia can be treated surgically either by performing either a frenectomy or a frenotomy. Although the process of lingual frenectomy is relatively straightforward, the anatomical location and structure of the lingual tissue make it susceptible to various intraoperative and postoperative complications. These complications fall into two categories: immediate complications occurring within hours of surgery and delayed complications arising days or weeks later. Immediate complications may include excessive bleeding from injuries to nearby blood vessels, the formation of retention cysts or ranulas due to blockage of Wharton’s duct, and sublingual hematomas from blood leaks into the tissue. Delayed complications can involve infections in the sublingual and submandibular spaces, often due to contamination from deep incision errors, infected sutures, or compromised immune systems. There is also a risk of reattachment or recurrence of frenal tissue due to scar formation or insufficient incisions. Additional issues may include new or worsening speech disorders from limited tongue movement due to scar tissue. Numbness and tingling in the tongue and surrounding soft tissues may result from nerve injury, pressure from healing tissues, swelling, or the effects of anesthesia [10].

In this case report, the patient developed a hematoma following a lingual frenectomy. An oral hematoma is a collection of blood that pools outside of blood vessels in the mouth, which can arise from trauma or injury to blood vessels during surgery. This condition can result in severe airway obstruction and may become life-threatening. The main etiology of excessive intraoperative bleeding following a frenectomy is an accidental injury to the major or minor blood vessels (such as the submental or sublingual artery) while excising the aberrant frenal attachment. Around 3%-8% of episodes of bleeding are observed in clinical practice during or after a frenectomy. Therefore, a deep and long incision that extends beyond the tongue into the gingival or mucosal tissue on the lingual aspect of the anterior mandible should be avoided to prevent injury to the branches of the inferior alveolar canal and its anastomosing plexus. One more effective way to prevent hematoma is to ensure hemostasis by applying a pressure pack with local hemostatic agents such as an absorbable collagen sponge, oxidized cellulose, hemocoagulase, topical thrombin, etc., and achieve primary closure. It is also recommended to pre-suture the base of the tongue with interrupted sutures at the most coronal and apical extension of the frenum to reduce the risk of bleeding [11]. This prevents any secondary bleeding, which could lead to hematoma formation. This condition can result in severe airway obstruction and may become life-threatening. One way to manage the hematoma after a lingual frenectomy includes the administration of intravenous antibiotics and analgesics [12], along with applying a magnesium sulfate dressing. Surgical intervention is only considered in severe cases with a compromise of the airway. In such situations, immediate medical attention is crucial due to the potential for airway obstruction.

Our findings align with the case of an infected sublingual hematoma described by Isaiah and Pereira which occurred after a lingual frenectomy and was treated with intravenous antibiotics [13]. Furthermore, Solis-Pazmino et al. highlighted a significant gap in reporting complications following tongue-tie release in their systematic review of major complications after frenotomy. It raises concerns about the low incidence of complications being adequately tracked and reported across different specialties [14]. Hence, researchers should be encouraged to report various complications encountered during routine dental care along with a note on its management.

Prevention of complications following a lingual frenectomy

Precise incisions, minimal removal of frenal tissue, and thorough suturing are critical to avoid any postoperative complications following lingual frenectomy. Care should be taken to avoid trauma to vital structures like salivary gland ducts and blood vessels. Postoperative observation should be scheduled after 24 hours to check for any bleeding tendency and hematoma development. Proper post-surgical instructions to the patient and parents and follow-up can minimize the complications following a lingual frenectomy. Reattachment of the frenum can be evaded by performing tongue exercises, observations, and immediate reporting to the clinician.

## Conclusions

Tongue-tie can significantly impair a patient's ability to move their tongue, resulting in speech difficulties. Timely intervention, often through surgical procedures like lingual frenectomy, is essential. While lingual frenectomy is common, it is highly technique-sensitive, and the unique anatomy of the lingual frenum makes it vulnerable to both intraoperative and postoperative complications. This case report highlights the clinical presentation of hematoma after 24 hours followed by lingual frenectomy and the management of the same. Early diagnosis and management of postoperative hematomas are crucial to prevent serious issues such as airway obstruction. To manage such risks effectively, a comprehensive understanding of potential complications associated with lingual frenectomy and their management is essential, warranting clinicians to be updated regularly. Additionally, improved documentation and reporting of complications are needed to refine clinical protocols and outcomes.
